# Agreement was moderate between data-based and opinion-based assessments of biases affecting randomized trials within meta-analyses

**DOI:** 10.1016/j.jclinepi.2020.05.009

**Published:** 2020-09

**Authors:** Rebecca M. Turner, Kirsty M. Rhodes, Hayley E. Jones, Julian P.T. Higgins, Jessica A. Haskins, Penny F. Whiting, Asbjørn Hróbjartsson, Deborah M. Caldwell, Richard W. Morris, Barnaby C. Reeves, Helen V. Worthington, Isabelle Boutron, Jelena Savović

**Affiliations:** a MRC Clinical Trials Unit, University College London, London, UK; b MRC Biostatistics Unit, School of Clinical Medicine, University of Cambridge, Cambridge, UK; c Statistical Innovation, Oncology Biometrics, AstraZeneca, Cambridge, UK; d Population Health Sciences, Bristol Medical School, University of Bristol, Bristol, UK; e Centre for Evidence-Based Medicine Odense (CEBMO), Odense University Hospital, Odense, Denmark; f Department of Clinical Research, University of Southern Denmark, Odense, Denmark; g Open Patient data Explorative Network (OPEN), Odense University Hospital, Odense, Denmark; h Clinical Trials and Evaluation Unit, Bristol Trials Centre, Bristol Medical School, University of Bristol, Bristol, UK; i Division of Dentistry, School of Medical Sciences, University of Manchester, Manchester, UK; j Centre d’Épidémiologie Clinique, Hôpital Hôtel-Dieu, Assistance Publique Hôpitaux de Paris, Paris, France; k Team METHODS, Centre of Research in Epidemiology and Statistics–CRESS Inserm UMR1153, Paris, France; l Université Paris Descartes, Paris, France; m NIHR Applied Research Collaboration (ARC) West, University Hospitals Bristol NHS Foundation Trust, Bristol, UK

**Keywords:** Meta-analysis, Systematic reviews, Randomized trials, Bias

## Abstract

**Background and Objective:**

Randomized trials included in meta-analyses are often affected by bias caused by methodological flaws or limitations, but the degree of bias is unknown. Two proposed methods adjust the trial results for bias using empirical evidence from published meta-epidemiological studies or expert opinion.

**Methods:**

We investigated agreement between data-based and opinion-based approaches to assessing bias in each of four domains: sequence generation, allocation concealment, blinding, and incomplete outcome data. From each sampled meta-analysis, a pair of trials with the highest and lowest empirical model-based bias estimates was selected. Independent assessors were asked which trial within each pair was judged more biased on the basis of detailed trial design summaries.

**Results:**

Assessors judged trials to be equally biased in 68% of pairs evaluated. When assessors judged one trial as more biased, the proportion of judgments agreeing with the model-based ranking was highest for allocation concealment (79%) and blinding (79%) and lower for sequence generation (59%) and incomplete outcome data (56%).

**Conclusion:**

Most trial pairs found to be discrepant empirically were judged to be equally biased by assessors. We found moderate agreement between opinion and data-based evidence in pairs where assessors ranked one trial as more biased.

**What is new?**

**Key findings**
• We found moderate agreement between opinion- and data-based evidence in the rankings of pairs of randomized trials by bias severity, in pairs where assessors ranked one trial as more biased.
• Most trial pairs found to be discrepant empirically under a bias model fitted to meta-epidemiological data were judged to be equally biased by assessors.

**What this adds to what was known?**
• Methods for bias adjustment in meta-analysis have been proposed by a number of authors and are usually informed by empirical evidence or elicited expert opinion on bias.
• The extent to which assessors' opinions on bias are similar to empirical estimates informed by meta-epidemiological research has not previously been evaluated.
• Bias adjustment can be informed by a combination of empirical evidence and opinion, with the aim of reducing uncertainty by using knowledge of the specific studies included in a meta-analysis.

**What is the implication and what should change now?**
• Our finding that most trial pairs were ranked as equally biased suggests that incorporating opinion on bias may not reduce uncertainty much, compared with using empirical distributions for bias alone.

## 1 Introduction

A meta-analysis of the results from relevant randomized trials is often regarded as the best evidence evaluating the effectiveness of a health care intervention [1]. Meta-analysis results summarize the findings from multiple studies and are more precise and usually more influential than the results from a single trial. Their findings inform public health policy decisions made by organizations such as the National Institute for Health and Care Excellence, as well as health care decisions made by individual patients, doctors, and institutions. Randomized trials vary in methodological quality, and flaws in the trial conduct can lead to biased estimation of the intervention effect [2]. If a meta-analysis makes no allowance for methodological flaws, there is a danger that the results could be biased and more precise than they should be [3], which can lead to inappropriate health care decisions.

Randomized trials should use rigorous methods that minimize the risk of bias and preserve comparability of the intervention groups. For example, concealment of randomized allocation ensures that the order of assignments to intervention groups cannot be predicted in advance and thereby removes the influence of patient characteristics on the probability of assignment to a group. Blinding of participants and caregivers to randomized allocation prevents differences in patient management between groups and blinding of outcome assessors (including participants when outcomes are reported by them) prevents knowledge of allocation influencing outcome measurement. Inadequacies in allocation concealment and blinding have been found to be associated with exaggeration of intervention effects [[4], [5], [6], [7], [8]]. Meta-analyses often include trials that vary in methodological adequacy with respect to these characteristics and others.

Assessing the risk of bias in included studies is a mandatory step in a systematic review [9,10], but there is no established method for combining bias assessments with a meta-analysis to guide interpretation of the effect of an intervention. Most systematic reviews do not incorporate bias assessments into the statistical analysis [11]. In those which do incorporate bias assessments, the most common approach is to perform a sensitivity analysis excluding high-risk studies, after a primary analysis including all evidence. This is problematic because it requires researchers to categorize available trials as either “good” and eligible for inclusion or “bad” and to be excluded. In many meta-analyses, a criterion to dichotomize trials as good or bad is not easily chosen, and if few trials remain eligible for inclusion, precision could be greatly reduced. For example, 43% of trials were judged to be at high risk of bias for at least one domain of the Cochrane Risk of Bias Tool [12], so exclusion on this basis could almost halve the number of trials included. Under this approach to addressing biases, discarded trials are regarded as providing no useful information at all, whereas included trials are implicitly assumed to be unaffected by within-trial biases. Most meta-analyses include trials that lie somewhere between these two extremes. Although sensitivity analyses based on the risk of bias are often reported, decision-making will usually be based on a single summary result, and it would therefore be desirable for the primary meta-analysis to incorporate adjustment for within-trial biases. Adjusting a meta-analysis for biases that are present in included trials is often considered controversial. However, the conventional approach of making no adjustment to the results even when potential causes of bias are present in a trial is equivalent to assigning an extremely strong opinion to the assumption that the bias is equal to zero.

Methods for bias adjustment in meta-analysis have been proposed by a number of authors, allowing the influence of evidence from less rigorous trials to be reduced in the combined analysis [3,[13], [14], [15], [16], [17]]. Although the potential causes of bias are often known, the impact of bias affecting each trial is unknown. Distributions describing the expected level of within-trial bias and the uncertainty about the bias are constructed from external evidence, which is typically in the form of an expert opinion or relevant empirical data. Empirical evidence on biases affecting randomized trials is available from meta-epidemiological studies that analyze large numbers of meta-analyses to examine the association between trial design characteristics and trial results [18]. Meta-epidemiological research has provided evidence on the biases associated with flaws in sequence generation, allocation concealment, blinding, and incomplete outcome data [[4], [5], [6],[19], [20], [21]]. Welton et al. [17] proposed a method that uses generic empirical evidence on the magnitude of biases, obtained from meta-epidemiological studies based on collections of meta-analyses. Turner et al. [16] proposed a method that uses elicited expert opinion on the likely magnitude of biases, informed by detailed assessment of the trials in the meta-analysis. The extent to which assessors’ opinions on bias are similar to empirical estimates informed by meta-epidemiological research has not previously been evaluated.

In some instances, it would be desirable for bias adjustment in meta-analysis to be informed by a combination of empirical evidence on bias and opinion. For example, available meta-epidemiological evidence may be considered only partially relevant to a specific meta-analysis because of a difference in population or intervention settings, and expert opinion could be used to adjust the data-based distribution for bias to the target setting. If relying on meta-epidemiological evidence alone, the predicted distribution for within-trial bias is often very imprecise because it allows for variability in bias across the collection of meta-analyses. By using opinion informed by knowledge of the studies included in a meta-analysis, it is likely that this uncertainty can be reduced. Using a combination of data-based evidence and opinion for the reasons described previously would be considered more valid if these approaches were known to produce similar estimates for bias.

In this research, we obtain opinions on the bias associated with four domains, using meta-analyses sampled from a meta-epidemiological study. Our aims were to examine agreement among experts and subsequently to explore agreement between empirical data-based and opinion-based approaches to assessing bias.

## 2 Methods

### 2.1 Outline of our approach

The approach to adjusting for biases based on empirical evidence involves fitting a hierarchical model to the data from trials included in each of a collection of meta-analyses [17]. For our investigations, we used data from the Risk of Bias in Evidence Synthesis (ROBES) study [6]. Within each meta-analysis extracted from the ROBES database, we selected the two trials with the highest and lowest model-based bias estimates, and then elicited an opinion on which trial was judged to be more biased. We examined agreement between model-based and opinion-based estimates of bias within selected pairs of trials.

### 2.2 ROBES study

The ROBES database consists of meta-analyses extracted from the April 2011 issue of the Cochrane Database of Systematic Reviews, in which Cochrane review authors had implemented the “risk of bias” tool to assess potential biases in included trials [22]. The ROBES study [6] included 228 meta-analyses in total, from Cochrane reviews that reported information on all five recommended risk of bias domains: sequence generation, allocation concealment, blinding, incomplete outcome data, and selective outcome reporting. Review authors had recorded whether there was a low, high, or unclear risk of bias in each bias domain, together with comments or quotes from the trial publication to justify each judgment. Meta-analyses were excluded if they included fewer than five trials or if a summary estimate was not reported in the review (for example, because pooling was considered inappropriate). One or more binary outcome meta-analyses (with sets of included trials that were unique to each meta-analysis) from each eligible review were included in the ROBES database; primary outcomes were chosen where possible [6].

### 2.3 Selection of pairs of trials within meta-analyses

For each meta-analysis, we selected a pair of trials with the highest and lowest model-based bias estimates, representing the least and the most biased trials among those included in the meta-analysis, for each of four bias domains: allocation concealment, sequence generation, blinding, and incomplete outcome data. These pairs were selected to present them to expert assessors, asking them which trial of each pair they judged to be at the greatest risk of bias in each domain examined. The process of selecting pairs of trials is described in detail as follows.

For each bias domain in turn, we first sampled 30 meta-analyses from the ROBES study. Meta-analyses included in the ROBES study were sampled from the Cochrane Database of Systematic Reviews in April 2011. Meta-analyses were sampled from the set of meta-analyses including at least one trial judged to be at low risk of bias and at least two trials judged to be at high or unclear risk of bias. A trial at low risk was needed as a comparator, to enable bias estimates to be obtained for trials with high- or unclear-risk judgments; at least two of the latter were required in order that the two with the highest and lowest bias estimates could be selected. For example, when sampling meta-analyses to examine the bias associated with allocation concealment, we sampled 30 meta-analyses including at least one trial assessed by review authors to have adequate allocation concealment and at least two trials assessed to have inadequate or unclear allocation concealment. To ensure that different outcome types were represented, each set of 30 meta-analyses comprised randomly selected samples of 15 eligible meta-analyses with outcomes judged to be objective or semiobjective (“objectively ascertained but potentially influenced by judgment”) in the ROBES study and 15 eligible meta-analyses with outcomes judged to be subjective or of mixed types within the meta-analysis [6]. The choice of sample size of 30 meta-analyses per bias domain is justified in the Appendix.

For each bias domain in turn, we fitted the bias model proposed by Welton et al. to all meta-analyses in the ROBES database and obtained estimates (together with uncertainty) for the trial-specific biases within the 30 sampled meta-analyses. The binary outcome data $r_{mia}$ and $n_{mia}$ (representing the number of events and total number of subjects) from each trial arm *a* of the trial *i* within the meta-analysis *m* were assumed to have a binomial likelihood, $r_{mia}∼Bin\left(p_{mia},n_{mia}\right)$. The following hierarchical bias model includes effects of trial-specific biases $\beta_{mi}$ associated with a known trial characteristic $Z_{mi}$ and allows for within–meta-analysis bias variation $\kappa^{2}$ and between–meta-analysis bias variation $\phi^{2}$ [17]. Treatment effects $\delta_{mi}$ are assumed random across trials within meta-analyses, with separate between-trial heterogeneity variances $\tau_{m}^{2}$. The values of $\delta_{mi}$ and $\tau_{m}^{2}$ were assumed to be unrelated across meta-analyses.

(1) $$\begin{array}{c} \text{logit}\left(p_{mia}\right)=\mu_{mi}+X_{mi}\left(\delta_{mi}+\beta_{mi}Z_{mi}\right)\\ \delta_{mi}\;∼\;N\left(d_{m},\tau_{m}^{2}\right)\\ \beta_{mi}\;∼\;N\left(b_{m},\kappa^{2}\right)\\ b_{m}\;∼\;N\left(b_{0},\phi^{2}\right) \end{array}$$

Posterior mean values of the $\beta_{mi}$ were used as bias estimates and viewed as model-based assessments for the extent of bias in particular trials. These are shrinkage estimates of bias, based on borrowing information across the meta-analyses in the ROBES database.

Next, for each bias domain in turn and within each sampled meta-analysis, we selected the pair of trials with the highest and lowest bias estimates, among the trials with a judgment of high or unclear risk of bias. The selected pairs of trials from each of the sampled meta-analyses formed our study data set in which empirical data-based and opinion-based approaches to assessing bias were compared.

### 2.4 Elicitation of opinion on bias

Every trial in each pair was summarized by a description of the trial participants, interventions, outcomes, and methods (together with additional notes, if available), extracted from the study characteristics tables reported by Cochrane reviewers. Trial sample sizes were added to each trial design summary, but no treatment effect estimates were provided. Support text for the risk of bias judgments (without the actual judgments) was extracted from the Cochrane risk of bias tables for each trial and included in the summary information and checked against the original trial reports by the research team. If no support text was available in the risk of bias table or if it was incomplete, vague, or not directly relevant to the given bias domain, it was extracted from the trial reports by the research team.

We recruited six assessors (A.H., D.M.C., R.W.M., B.C.R., H.V.W., and I.B.) with expertise in clinical research methodology and evidence-based medicine, by personal invitation. For each trial pair, assessors were given information packs (see example in Appendix) and asked to complete them independently. In total, each trial pair was assessed three times, by three of six assessors. Trials within the pairs were labeled “trial A” and “trial B″ at random. For each of the four bias domains (sequence generation, allocation concealment, blinding, and incomplete outcome data), the assessors were asked to choose between the following three judgments: “trial A is more biased,” “trial B is more biased,” or “trial A and trial B are equally biased.” We note that assessors were asked to make judgments for all four bias domains, without knowledge of the bias domain for which the trial pair had been selected. In addition, assessors were asked to choose between the same three judgments with respect to the overall risk of bias. Alongside each judgment, assessors were asked to provide a rating from 1 to 5 for their confidence in that judgment, where 1 represents “not at all confident” and 5 represents “very confident.” The assessors attended a 1-day meeting to carry out their rankings and were asked not to discuss their judgments with other assessors; several assessors required more time and completed the work later on.

### 2.5 Data analysis

We examined agreement in the trial pair rankings (ordering of trials A and B with respect to extent of bias) among the bias assessors, using unweighted kappa statistics and 95% confidence intervals. Analyses were performed for each bias domain separately and then for all bias domains combined, using rankings from all trial pairs in the study data set.

We assessed agreement between the trial pair rankings produced by assessors and the ranking based on estimated biases from the bias model. We reported the proportion of trial pairs in which assessors chose one trial as more biased (rather than saying they were equally biased). Of the judgments in which one trial was believed to be more biased than the other, we calculated the proportion in which assessor opinion agreed with the model-based ranking of the trials. Analyses were performed for each bias domain separately, using the rankings from the subset of 30 meta-analyses sampled for that bias domain.

Next, we conducted exploratory multinomial logistic regression analyses to examine the association between assessor opinions and model-based differences in bias estimates between the trials in each pair. We used regression to explore whether agreement between assessor ranking and model-based ranking was associated with the magnitude of the difference in estimated biases for each trial pair. For each combination of trial pair (*i*) and assessor (*j*), there are three possible outcomes: disagreement between the assessor and model-based rankings, agreement between the assessor and model-based rankings, or assessors ranking trials as equally biased. Disagreement between the assessor and model-based ranking was treated as the baseline category (*k* = 0) for the response variable, and a multinomial logistic regression model was created to estimate the odds ratio for each of the two alternative categories: assessors ranking trials as equally biased (*k* = 1) and assessors agreeing with the model-based ranking (*k* = 2). As a single covariate in the model, we included the magnitude of difference in bias estimates in the trial pair. The multinomial logistic regression model was

(2) $$\log it\left(\pi_{ijk}\right)=\alpha_{k}+\beta_{k}x_{i}+u_{ik}+\gamma_{jk}$$

Where, $\pi_{ijk}$ represents the probability of outcome category *k* for assessor *j* in trial pair *i*, and $x_{i}$ is the model-based difference in bias estimates (calculated as the difference between the most extreme and least extreme bias values). To allow for similarity in judgments on the same trial pair (or equivalently, variation between trial pairs), we included a random intercept $u_{i}$ for each of the 30 trial pairs. We also included a fixed effect $\gamma_{j}$ for each of the six different assessors. We focus on the regression coefficient $\beta_{2}$ of the model-based difference in bias estimates. A positive value for this coefficient indicates that, on average, assessor agreement with model-based rankings is associated with the magnitude of the estimated difference in bias from the model.

All regression models were fitted using MCMC methods within WinBUGS [23] (see Appendix).

## 3 Results

### 3.1 Descriptive analyses

Our data set consisted of 101 trial pairs in total because there was some overlap between the sets of 30 meta-analyses sampled for each of the four bias domains. Table 1 summarizes the types of interventions and outcomes evaluated in the sampled meta-analyses. Most (64%) of sampled meta-analyses corresponded to pharmacological vs. placebo/control comparisons, whereas 25% were nonpharmacological vs. control comparisons, and the remainder represented comparisons of two active treatments. Objective outcomes were evaluated in 36% of sampled meta-analyses overall, 16% evaluated semiobjective (“objectively ascertained but potentially influenced by judgment”) outcomes, and 46% evaluated subjective outcomes. The median number of trials included in the meta-analyses was 13 (interquartile range (IQR) 9 to 24). Meta-analysis characteristics were fairly similar across the meta-analysis samples selected for each bias domain (Table 1).

**Table 1:** Characteristics of meta-analyses sampled from the ROBES data set, for each bias domain and overall

Characteristics of meta-analyses sampled	Bias domain				
	Sequence generation (*n* = 30)	Allocation concealment (*n* = 30)	Blinding (*n* = 30)	Incomplete outcome data (*n* = 30)	Overall (*n* = 101)
Type of intervention comparison					
Pharmacological vs. placebo/control	17 (57%)	21 (70%)	19 (63%)	20 (67%)	65 (64%)
Pharmacological vs. pharmacological	5 (17%)	1 (3%)	1 (3%)	2 (7%)	8 (8%)
Nonpharmacological vs. placebo/control	8 (27%)	7 (23%)	8 (27%)	7 (23%)	25 (25%)
Nonpharmacological vs. nonpharmacological	0 (0%)	1 (3%)	2 (7%)	1 (3%)	3 (3%)
Type of outcome measure					
Objective	11 (37%)	11 (37%)	10 (33%)	11 (37%)	36 (36%)
Semiobjective	5 (17%)	4 (13%)	5 (17%)	3 (10%)	16 (16%)
Subjective	13 (43%)	14 (47%)	14 (47%)	15 (50%)	46 (46%)
Mixed types within the meta-analysis	1 (3%)	1 (3%)	1 (3%)	1 (3%)	3 (3%)
Number of trials: median (interquartile range)	13.5 (10 to 20)	13.5 (9 to 24)	12 (8 to 18)	15 (9 to 24)	13 (9 to 24)

Table 2 shows the frequencies of the risk of bias profiles (combinations of the risk of bias judgments for the four bias domains, reported by Cochrane authors) among the trials selected as having the lowest or highest bias estimates within meta-analyses. Of 202 trials, 120 (59%) had judgments of high or unclear risk of bias for three or four bias domains, and no trials had low risk of bias judgments for all domains. Differences within trial pairs are summarized in Table 3. The risk of bias judgments differ within pairs for only one bias domain or no bias domains in 59 of 101 trial pairs, and differ for all four bias domains in only 4 of 101 pairs.

**Table 2:** Frequencies of the risk of bias profiles (from Cochrane reviews) in trials selected from sampled meta-analyses

Bias domain				Frequency (%) (*n* = 202)
SG	AC	B	IOD	
				0 (0%)
				0 (0%)
				6 (3%)
				13 (6%)
				7 (3%)
				20 (10%)
				7 (3%)
				3 (1%)
				8 (4%)
				7 (3%)
				11 (5%)
				34 (17%)
				20 (10%)
				7 (3%)
				5 (2%)
				54 (27%)

*Abbreviations:* SG, sequence generation; AC, allocation concealment; B, blinding; IOD, incomplete outcome data.

—high/unclear risk of bias.

—low risk of bias.

**Table 3:** Differences in the risk of bias profiles (from Cochrane reviews) within trial pairs

Extent of difference in judgments within trial pairs	Frequency (%) (*n* = 101)
High/unclear/low judgments match for all bias domains	23 (23%)
Difference in judgments for one bias domain	36 (36%)
Differences in judgments for two bias domains	27 (27%)
Differences in judgments for three bias domains	11 (11%)
Differences in judgments for four bias domains	4 (4%)

Table 4 describes the extent of agreement among the bias assessors when judging which trial of each pair they believed to be more biased, showing the estimated kappa statistics in the rankings of the three assessors. There was fair to moderate agreement among the rankings. For sequence generation, the percentage of pairs in which all three assessments agreed was 50% and the kappa statistic was 0.43 (95% CI: 0.37 to 0.50). For allocation concealment, the percentage in which all three assessors were in agreement was 56% and the kappa statistic was 0.46 (95% CI: 0.40 to 0.52). There was moderate agreement among rankings for blinding; the percentage agreement across all three assessors was 60% and kappa was estimated as 0.45 (95% CI: 0.39 to 0.51). There was less agreement among assessors for incomplete outcome data; the percentage in which all three assessors agreed was 31% and the kappa statistic was 0.21 (95% CI: 0.14 to 0.27). For overall risk of bias, the percentage of trial pairs in which all three assessors agreed was 32% and the kappa statistic was 0.26 (95% CI: 0.19 to 0.32).

**Table 4:** Kappa statistics with 95% confidence intervals for assessing agreement in rankings among the three bias assessors

Bias domain	Trial pairs	Unweighted kappa (95% CI)	Interpretation	% Trial pairs with three assessments in agreement
Sequence generation	All 101	0.43 (0.37 to 0.50)	Moderate agreement	50/101 (50%)
Allocation concealment	All 101	0.46 (0.40 to 0.52)	Moderate agreement	57/101 (56%)
Blinding	100^a^	0.45 (0.39 to 0.51)	Moderate agreement	60/100 (60%)
Incomplete outcome data	99^a^	0.21 (0.14 to 0.27)	Fair agreement	31/99 (31%)
Overall	97^a^	0.26 (0.19 to 0.32)	Fair agreement	31/97 (32%)

a Missing expert opinions.

**Fig. 1:**
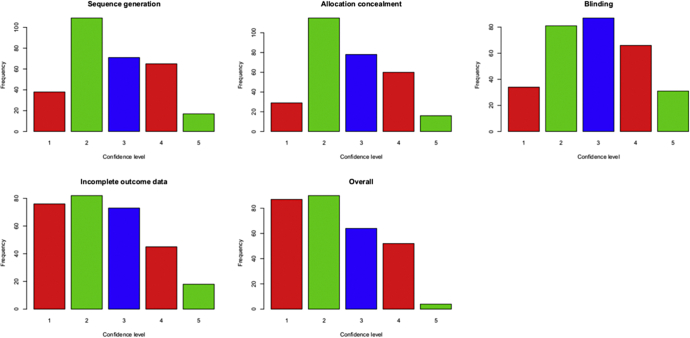
The confidence of assessors in their opinions on each bias domain and overall bias, where 5 represents “very confident” and 1 represents “not at all confident.”

The assessors specified a confidence level of 1 (not at all confident) to 5 (very confident) about their opinion. We summarize the confidence levels in Figure 1. Assessor confidence levels were comparable for sequence generation, allocation concealment, and blinding. For each of these bias domains, the median confidence level across all trial pairs and all assessors was 3 (IQR: 2 to 4). Confidence levels tended to be lower for incomplete outcome data and for overall bias (median 2, IQR: 1 to 3 for each). Confidence levels were no higher when examined only in the bias domain for which the trial pair had been selected.

For each bias domain, 30 trial pairs were ranked by each of three assessors, resulting in 90 assessor opinions. For sequence generation, 36 (40%) of the 90 assessor opinions ranked one trial as more biased than the other (Table 2). For allocation concealment, blinding, and incomplete outcome data, respectively, 14 (16%), 24 (27%), and 41 (46%) opinions ranked one trial as more biased. Table 5 reports the proportion of assessor opinions that agreed with the model-based ranking of trial pairs. Among the assessor opinions that judged one trial as more biased (rather than trials equally biased), the proportion that agreed with the ranking based on the bias model was high for allocation concealment (79%) and blinding (79%). For sequence generation and assessment of incomplete outcome data, agreement was lower at 59% and 56%, respectively (i.e., not much better than chance).

**Table 5:** Frequency of assessor opinions ranking one trial as more biased (rather than choosing trials equally biased)

Bias domain	How often did the assessors choose one trial as more biased (rather than equally biased)?	Of those that chose one trial as more biased, what proportion agreed with the model?
Sequence generation	36/90 (40%)	23/36 (59%)
Allocation concealment	14/90 (16%)	11/14 (79%)
Blinding	24/90 (27%)	19/24 (79%)
Incomplete outcome data	41/90 (46%)	23/41 (56%)

Of those that chose one trial as more biased, we report the proportion that agreed with the fitted model of Welton et al.

### 3.2 Regression analyses

In the exploratory multinomial logistic regression analyses, we focus on the regression coefficient $\beta_{2}$ of the model-based difference in fitted bias (Table 6). Although this was estimated as positive for allocation concealment and incomplete outcome data, the 95% credible intervals were very wide and contained the null value, representing no association between the magnitude of difference in model-based bias estimates and agreement between assessor and model-based rankings. For sequence generation and blinding, the regression coefficient was estimated as negative, again with very wide 95% credible intervals containing the null value. Similarly, we cannot conclude whether smaller differences in model-based bias estimates were associated with assessors ranking trials as equally biased. There is insufficient information in the data for us to be able to draw any conclusions from the results (Table 6); all intervals for model parameters were wide and close to the ranges of the assigned prior distributions.

**Table 6:** Results from the exploratory multinomial regression to examine the association between assessor opinion and model-based difference in bias estimates: central parameter estimates (95% credible intervals)

Outcome	Model parameter	Sequence generation	Allocation concealment	Blinding	Incomplete outcome data
Assessor and model-based rankings agree	Model-based difference in bias estimates^a^$\left(\beta_{2}\right)$	−0.07 (−6.25 to 6.03)	0.04 (−6.20 to 6.23)	−0.08 (−6.32 to 6.08)	0.42 (−5.80 to 6.60)
	Assessor effects $\left(\alpha_{2}+\;\gamma_{j2}\right)$				
	1	−0.77 (−2.95 to 1.06)	−1.21 (−4.67 to 1.79)	0.08 (−4.07 to 4.11)	−1.47 (−4.44 to 1.03)
	2	N/A	−2.50 (−7.61 to 2.08)	1.98 (−0.04 to 4.38)	0.10 (−1.90 to 1.86)
	3	−0.52 (−3.88 to 2.80)	1.24 (−1.08 to 3.84)	−1.20 (−4.66 to 1.95)	−0.22 (−2.42 to 1.74)
	4	0.27 (−2.14 to 2.61)	−0.35 (−4.39 to 3.52)	0.46 (−1.59 to 2.40)	−0.02 (−3.22 to 3.05)
	5	1.32 (−1.66 to 4.50)	−0.86 (−4.29 to 2.18)	−1.76 (−6.94 to 2.64)	0.37 (−1.70 to 2.39)
	6	1.59 (−0.93 to 4.34)	1.05 (−2.22 to 4.39)	0.50 (−2.82 to 3.93)	−0.13 (−2.35 to 1.99)
	Between trial–pair standard deviation	3.31 (1.43 to 4.88)	2.43 (0.06 to 4.70)	2.17 (0.36 to 4.47)	2.35 (0.82 to 4.52)
Assessor and model-based rankings disagree	Baseline outcome				
Trials equally biased	Model-based difference in bias estimates^b^$\left(\beta_{1}\right)$	−0.23 (−6.47 to 6.05)	−0.37 (−6.57 to 5.86)	−0.48 (−6.61 to 5.64)	0.14 (−6.07 to 6.23)
	Assessor effects $\left(\alpha_{1}+\;\gamma_{j1}\right)$				
	1	0.80 (−1.12 to 2.71)	3.80 (1.82 to 6.43)	3.02 (−0.27 to 7.03)	−0.59 (−3.00 to 1.51)
	2	N/A	5.14 (1.79 to 9.52)	2.80 (0.92 to 5.13)	1.11 (−0.45 to 2.76)
	3	1.10 (−2.47 to 4.61)	2.59 (0.51 to 5.00)	2.67 (0.58 to 5.37)	2.04 (0.53 to 3.90)
	4	2.62 (0.52 to 4.99)	4.67 (1.86 to 8.50)	1.10 (−0.84 to 2.89)	1.99 (−0.36 to 4.76)
	5	1.86 (−0.89 to 4.89)	2.90 (0.76 to 5.56)	5.07 (2.20 to 9.22)	1.08 (−0.67 to 2.89)
	6	3.94 (1.43 to 6.85)	4.32 (1.78 to 7.57)	4.76 (2.20 to 8.10)	0.94 (−0.91 to 2.90)
	Between-trial–pair standard deviation	3.99 (2.21 to 4.95)	2.03 (0.41 to 4.49)	1.84 (0.37 to 4.20)	1.94 (0.59 to 3.90)

a A positive value for $\beta_{2}$ indicates that, on average, greater differences in estimated bias within trial pairs are associated with assessor rankings agreeing with the model-based rankings.

b A positive value for $\beta_{1}$ indicates that, on average, greater differences in estimated bias within trial pairs are associated with assessor ranking trials as equally biased.

## 4 Discussion

Agreement between opinion-based and model-based rankings of bias magnitude was high for sequence generation and allocation concealment and moderate for blinding and incomplete outcome data, among trial pairs in which assessors ranked one trial as more biased. However, in most of trial pairs, assessors ranked trials as equally biased, although the two trials had been selected on the basis of having high and low bias estimates (within a given meta-analysis) under the bias model fitted. In these trial pairs, detailed trial descriptions did not lead assessors to judge the bias as higher in one trial than another. There was fair to moderate agreement in rankings across bias assessors. In exploratory regression analyses, uncertainty was too high for us to draw conclusions about associations between the magnitude of difference in model-based bias estimates and assessors agreeing with model-based rankings or assessors ranking trials as equally biased.

Published methods for bias adjustment in meta-analysis suggest making use of either empirical data-based evidence on biases or opinion on biases [3,13,[15], [16], [17]], but no previous comparison has been made between data-based distributions and assessors' opinions on bias. Access to a large collection of meta-analyses for which review authors have reported the risk of bias judgments and supporting information has enabled us to carry out a comparative study. We note that the empirical data-based distributions for bias were themselves informed indirectly by opinion because they were derived from a hierarchical model fitted to trial data within meta-analyses, in which judgment about each trial's risk of bias was used as a covariate. The model-based rankings rely on the appropriateness of the assumed model for the data and also on the risk of bias judgments reported by Cochrane reviewers. Reviewers follow the risk of bias protocols that aim to maximize reproducibility. It would not be possible to adjust a meta-analysis for trial-specific biases without incorporating some form of subjective judgment. Formal validation methods are not available for bias assessments because the true extent of bias in a given trial is unknown, but agreement between independent bias assessments would increase our confidence in them.

Because the actual magnitude of bias affecting the trial pairs selected from the sampled meta-analyses remains unknown, it is not possible to evaluate whether the data-based or opinion-based rankings are closer to the truth. Assessors indicated that their confidence in their own opinions on the rankings of trials within pairs was moderate or low. In our study, assessors were asked to carry out a large number of rankings during 1 day (although several assessors required more time and completed the work later on); the high workload may have affected their performance. When assessors are asked to provide opinions on biases affecting studies in a single meta-analysis, the number of studies assessed would typically be much smaller. We observed less agreement among assessors for incomplete outcome data than for the other bias domains. This may be related to the greater complexity of the bias in this domain, which depends on several factors, including the amount and distribution of missing data across intervention groups, the likely difference in the outcome between missing and nonmissing participants, and how the problem has been addressed in reported analyses [24]. We aimed to assess agreement among assessors pragmatically, so we did not attempt to increase interobserver agreement before carrying out the elicitation exercise.

In this work, opinions about biases were based on summary information about trials, informed primarily by the study characteristics and risk of bias tables reported by Cochrane reviewers and supplemented by additional information extracted from the trial reports by the research team. Assessors reported some difficulties in assessing bias on the basis of summary information and commented that for certain trials they would have liked access to the original trial publications. When eliciting opinions about within-trial biases, it might therefore be preferable to provide full publications, as Turner et al. did in their opinion-based method for bias adjustment [16], although this introduces some risk that assessments of bias are influenced by knowledge of the trial results unless all results are removed. Using all available sources of information (e.g., publication, statistical analysis plan, protocol, trial registration records etc.) is generally encouraged for assessing risk of bias in randomized controlled trials included in systematic reviews [25], to improve confidence in assessment. We were surprised that most of trial pairs were ranked as equally biased, and we suspect that the lack of detailed trial information contributed to this. We expect that differentiation between trials was reduced also by requesting categorical judgments for each trial pair rather than continuous judgments of bias (using a visual analog scale, for example) for each individual trial. Trials judged to be at a high or unclear risk of bias were grouped together in the hierarchical model used to estimate bias. Research has suggested that many trials judged to be at an unclear risk of bias for sequence generation and allocation concealment could be reclassified as low risk if information outside the trial publications was obtained [26]. Misclassification of the risk of bias judgments may have reduced or increased the differences within some of the selected trial pairs.

The risk of bias judgments are increasingly published for trials included in Cochrane reviews. It is desirable to incorporate these judgments about suspected biases into the statistical analyses performed and interpretation of the review findings [10,11]. The Cochrane database could in time provide extensive evidence on the degree of bias associated with combinations of the risk of bias judgments for different domains. In a separate article, we have explored methods for quantifying bias by using empirical distributions for the bias affecting trials with a specific set of risk of bias judgments, in combination with expert opinion [27]. However, our finding in this article that most of trial pairs were ranked as equally biased suggests that incorporating opinion on bias may not reduce uncertainty much, compared with using empirical distributions alone.

We found moderate agreement between opinion- and data-based evidence in the rankings of trial pairs by bias severity, in pairs where assessors ranked one trial as more biased. This finding provides some support for approaches combining data-based evidence with opinion on bias. However, trials were ranked as equally biased in most of trial pairs, indicating that trial summaries did not provide sufficient information to reach a ranking judgment.

## CRediT authorship contribution statement

**Rebecca M. Turner:** Conceptualization, Methodology, Investigation, Project administration, Writing - original draft. **Kirsty M. Rhodes:** Methodology, Investigation, Formal analysis, Writing - original draft. **Hayley E. Jones:** Conceptualization, Methodology, Investigation, Writing - review & editing. **Julian P.T. Higgins:** Conceptualization, Methodology, Investigation, Writing - review & editing. **Jessica A. Haskins:** Data curation, Writing - review & editing. **Penny F. Whiting:** Data curation, Writing - review & editing. **Asbjørn Hróbjartsson:** Resources, Writing - review & editing. **Deborah M. Caldwell:** Resources, Writing - review & editing. **Richard W. Morris:** Resources, Writing - review & editing. **Barnaby C. Reeves:** Resources, Writing - review & editing. **Helen V. Worthington:** Resources, Writing - review & editing. **Isabelle Boutron:** Resources, Writing - review & editing. **Jelena Savović:** Conceptualization, Methodology, Investigation, Project administration, Writing - review & editing.
