# Supplementary material for: Agreement was moderate between data-based and opinion-based assessments of biases affecting randomized trials within meta-analyses
Source: J Clin Epidemiol. 2020 Sep;125:16–25. doi: 10.1016/j.jclinepi.2020.05.009 (PMC7482431; doi:10.1016/j.jclinepi.2020.05.009)
Supplement: Appendix [file mmc1.docx]

## Appendix

### Sample size justification

Choice of sample size of 30 meta-analyses per bias domain was based on considering precision of estimation of the proportion of pairs for which model-based and opinion-based orderings of biases agree. Overlap between the sets of 30 meta-analyses sampled for different bias domains was expected, meaning that the total number of sampled meta-analyses was likely to be less than 120. In a sample of 90 trial pairs (assuming 25% reduction), the standard error for the proportion of pairs with agreement would be less than 0.05 (maximum value 0.049 for an observed proportion of 0.5), assuming a high between-assessor correlation of 0.8. In each bias domain sample of 30 trial pairs, the standard error would be less than 0.1.

### Model fitting

All regression models were fitted using MCMC methods within WinBUGS (23). We declared vague normal(0,10) priors for unknown regression coefficients and a uniform(0,5) prior for all standard deviations. Results were based on 100,000 iterations following a burn-in period of 10,000 iterations which was sufficient to achieve convergence. Convergence was checked using the Brooks-Gelman-Rubin statistic, as implemented in WinBUGS (23), with three chains starting from widely dispersed initial values.

### Example information pack for a trial pair

Instructions: The two trials laid out below are taken from the same meta-analysis. Please compare these two trials with respect to the magnitude of bias due to inadequacies in sequence generation, allocation concealment, blinding and incomplete outcome data.

**Review details**

Review ID: 736 CD number: CD005496

Review title: Probiotics for prevention of necrotizing enterocolitis in preterm infants

Participants/populations: Preterm infants < 37 weeks and/or birth weight < 2500 g.

Interventions: Enteral administration of any live microbial supplement (probiotics) at any dose for more than seven days compared to placebo or no treatment.

**Meta-Analysis details** Meta-analysis ID: 17134

Comparison details: Probiotics vs. Placebo or no treatment

Experimental intervention: Probiotics

Comparator intervention: Placebo or no treatment

Outcome: Severe necrotising enterocolitis (stage II-III)

**TRIAL A**

**Trial details** Trial ID: 21227 Trial name: Samanta 2009

| **Methods** | Prospective randomised double-blind control trial Method of generating randomisation sequence: Can't tell Allocation concealment: Can't tell Blinding of intervention: Can't tell Blinding of outcome measurement: Can't tell Complete follow-up: Yes |
| --- | --- |
| **Sample size** | 61 |
| **Participants** | Gestational age <32 weeks and VLBW infants (<1500 g) started feed enterally and survived beyond 48 h of life Demographic data: Probiotics Group N=91, gestational age 30.12 (weeks) (1.63), birth weight 1172 (143) Control Group N=95, gestational age 30.14 (weeks) (1.59), birth weight 1210 (143) |
| **Interventions** | The probiotic group received a probiotic mixture (Bifidobacteria infantis, Bifidobacteria bifidum, Bifidobacteria longum and Lactobacillus acidophilus, each 2.5 billion CFU) with expressed breast milk twice daily, the dosage being 125 g kg -1 till discharge. The control group was fed with breast milk only. |
| **Outcomes** | Feed tolerance in terms of days required to reach full enteral feeding Length of hospital stay NEC Sepsis Death due to NEC or sepsis |
| **Notes** | Neonatal Care Unit of Medical College and Hospital, Kolkata, India Period of study: October 2007 - March 2008 Published: 2009 Source of Funding: not specified in paper |

**Risk of bias table** Trial ID: 21227 Trial name: Samanta 2009

| **Bias Domain** | **Description of what was done (based on study reports/papers)** |
| --- | --- |
| Adequate sequence generation? | Quote: "infants were randomly assigned to two groups by random number table sequence" (p.129)  No further information provided. |
| Allocation concealment? | Not stated whether allocation was concealed prior to assignment  Paper states this is a prospective randomised double-blind control trial, but detail not provided. |
| Blinding? | Paper states this is a prospective randomised double-blind control trial, but detail not provided.  Quote: "the probiotic-fortified group received a probiotic mixture…with expressed breast milk daily…The control group was fed with breast milk only." (p.129)  Not stated whether mothers or personnel were aware of allocation.  Not stated whether assessors were blinded to allocation |
| Incomplete outcome data addressed? | Analysis appears to be based on full numbers of participants  Attrition and exclusions from analysis were not reported. |
| Free of selective reporting? | All 3 primary outcome measures reported (feed tolerance, length of hospital stay, morbidities). |
| Free of other bias? | Birth weight and gestational age were not significantly different between groups. No other statitistically significant demographic or clinical variables between groups.  Adverse effects not reported  No sample size calculations reported  Inclusion/exclusion criteria reported |

**TRIAL B**

**Trial details** Trial ID: 21228 Trial name: Sari 2010

| **Methods** | Single Center Method of generating randomisation sequence: Sequential numbers generated at the computer center of the NICU Allocation concealment: Can't tell Blinding of intervention: Can't tell Blinding of outcome measurement: Yes Complete follow-up: Yes |
| --- | --- |
| **Sample size** | 63 |
| **Participants** | Gestational age <33 weeks or birth weight <1500 g Demographic data: Probiotics Group N=110, gestational age 29.5 (weeks) (2.4), birth weight 1231 (262) Control Group N=111, gestational age 29.7 (weeks) (2.4), birth weight 1278 (282) |
| **Interventions** | VLBW infants who survived to start enteral feeding were randomised The study group were given L. sporogenes with a dose of 350.000.000 colony forming units added to breast milk or formula once a day starting with first feed until discharge. The control group were fed without L. sporogenes supplementation. |
| **Outcomes** | Death or severe NEC NEC (stage 2, 3, = 2) Death (attributable to NEC, not attributable to NEC) Total parental nutrition Intraventricular hemorrhage, grade 3-4, Sepsis (culture proven, gram negative, gram positive, fungus) NICU stay Feeding (amount, full feeding, intolerance) Weight gain |
| **Notes** | Turkey Period of study: October 2008 and June 2009 Published: Unpublished Source of Funding: not specified in paper |

**Risk of bias table** Trial ID: 21228 Trial name: Sari 2010

| **Bias Domain** | **Description of what was done (based on study reports/papers)** |
| --- | --- |
| Adequate sequence generation? | Quote: "The infants were randomly assigned to one of two groups prospectively. Randomization was performed by using sequential numbers generated at the computer center of the NICU" (p.435) |
| Allocation concealment? | Quote: "The allocations were contained in opaque, sequentially numbered sealed envelopes" (p.435) |
| Blinding? | Blinding of intervention:  Quote: "the only personnel who knew of the infants' group assignments were the investigators and those in the breast-milk team who were not involved in the care of the study infants" (p.435)  Supplementation given to the experimental group did not alter the appearance of the milk or formula.  Quote: "Fresh suspension of supplements were prepared by personnel in the breast-milk team who were not involved in the care of the infant and who followed instructions from the sealed envelope" (p.435)  Blinding of outcome:  Quote: "Whenever an infant was suspected to have NEC [outcome measure], the infant was evaluated by two senior-attending neonatologists who did not know the group assignment of the infant" (p.435) |
| Incomplete outcome data addressed? | Attrition and exclusions reported, no apparent imputation  Analysis only of sample after attrition (11 lost in experimental group, 10 lost in control). |
| Free of selective reporting? | Primary and secondary outcome measures were reported  Primary = death or stage >2NEC  Secondary = culture proven sepsis without NEC, intraventricular hemorrhage, feeding intolerance, feeding amount per week, days to reach full enteral feeding, wight gain per week. |
| Free of other bias? | Clinical and demographic characteristics did not differ between groups, except for:  Quote: " longer duration of umbilical venous catheterization in the probiotics group" (p.436)  Possible effect of this discussed in discussion  Inclusion/exclusion criteria reported  Adverse effects were reported.  Sample size calculations reported - number needed = 111 infants for each arm. Total sample = 110 experimental, 111 control  Quote: "the required sample size was above the actual numbers attained in our study, which in turn make the study underpowered to detect small differences" (p.438) |

**EXPERT VERDICT**

In which of these two trials would you expect inadequacies in each of the listed bias domains to cause greater bias towards overestimation of the effect estimate of treatment benefits for the experimental intervention?

Please consider each bias domain separately. For each bias domain, your decision should be explicit to that particular bias, but the whole risk of bias table may be taken into account (i.e. information provided under all other bias domain may influence your decision for any one particular domain).

Please tick only one verdict option per domain and indicate your level of confidence for each verdict on a scale of 1 to 5 (1 being not very confident at all and 5 being very confident).

Meta-analysis outcome: **Severe necrotising enterocolitis (stage II-III)**

|  | **Your verdict on bias**  Tick only one of the three possible options for each bias domain (each row) | | | **Confidence level score**  How confident are you about this verdict?  Enter confidence score between 1 and 5 for each bias domain, where 1 = not at all confident; 5 = very confident. |
| --- | --- | --- | --- | --- |
| **Bias domain**  (tick only one box per row for each domain & overall bias) | Option 1:  **Trial *A* is more biased** | Option 2:  **Trial *B* is more biased** | Option3:  **Trial A and Trial B are *equally* biased** |  |
| **Sequence generation** |  |  |  |  |
| **Allocation concealment** |  |  |  |  |
| **Blinding** |  |  |  |  |
| **Incomplete outcome data** |  |  |  |  |
| **Overall risk of bias for this outcome** |  |  |  |  |

**Expert Assessor notes *(optional)***

If you would like to add a reason for your decision please add it be
